# Evaluation of the Lung Immune Prognostic Index in Non-Small Cell Lung Cancer Patients Treated With Systemic Therapy: A Retrospective Study and Meta-Analysis

**DOI:** 10.3389/fonc.2021.670230

**Published:** 2021-06-24

**Authors:** Litang Huang, Hedong Han, Li Zhou, Xi Chen, Qiuli Xu, Jingyuan Xie, Ping Zhan, Si Chen, Tangfeng Lv, Yong Song

**Affiliations:** ^1^ Department of Respiratory and Critical Care Medicine, Affiliated Jinling Hospital, School of Medicine, Southeast University, Sch Med, Nanjing, China; ^2^ Department of Respiratory and Critical Care Medicine, Affiliated Jinling Hospital, Medical School of Nanjing University, Nanjing, China; ^3^ Department of Critical Care Medicine, Zhongda Hospital, School of Medicine, Southeast University, Nanjing, China; ^4^ Department of Ophthalmology, Affiliated Jinling Hospital, School of Medicine, Southeast University, Sch Med, Nanjing, China

**Keywords:** non-small cell lung cancer, immunotherapy, chemotherapy, targeted therapy, lung immune prognostic index (LIPI)

## Abstract

The lung immune prognostic index (LIPI) has been shown to be an important prognostic marker for various tumors. However, the prognostic value of LIPI among non-small cell lung cancer (NSCLC) patients treated with systemic therapy remains controversial. We aimed to evaluate survival status according to LIPI among NSCLC patients receiving different forms of systemic therapy at our institution. We also performed a meta-analysis of articles from PubMed and Embase to illustrate this question. For our cohort, we found that good LIPI was associated with better overall survival (OS) among 91 patients on immunotherapy, 329 patients on targeted therapy, and 570 patients on chemotherapy. For the meta-analysis, a total of eight studies with 8,721 patients were included. Pooled results showed that a higher LIPI (those with 1 or 2 factors) was associated with poor overall progression-free survival (PFS) (hazard ratio [HR], 1.57; 95% confidence interval [CI], 1.45−1.71) and OS (HR, 2.01; 95% CI, 1.75−2.31). Subgroup analyses showed that a higher LIPI was related to poor survival among patients prescribed different systemic therapies: immunotherapy (OS HR, 2.50; 95% CI, 1.99–3.13; PFS HR, 1.77; 95% CI, 1.56–2.01), chemotherapy (OS HR, 1.58; 95% CI, 1.34–1.86; PFS HR, 1.38; 95% CI, 1.23–1.55), and targeted therapy (OS HR; 2.15, 95% CI, 1.57–2.96; PFS HR, 1.60; 95% CI, 1.25–2.06). The study shows that the LIPI is a clinically significant prognostic factor for NSCLC patients receiving systemic therapy.

**Systematic Review Registration:**

https://www.crd.york.ac.uk/PROSPERO/, identifier CRD420209009.

## Introduction

Lung cancer is a common malignancy with high morbidity and mortality (1). More than 80% of lung cancer patients present with non-small cell lung cancer (NSCLC), and the majority of NSCLC patients are initially ascertained at an advanced disease stage (2). In addition to traditional chemotherapy, precision medicine has brought new therapeutic strategies for the treatment of NSCLC, such as targeted therapy and immunotherapy; these treatment modalities have achieved significant survival benefits in NSCLC patients (3–5). However, a considerable proportion of patients do not benefit from systemic therapy, including chemotherapy, targeted therapy, and immunotherapy (6, 7). Thus, researchers have investigated effective prognostic factors of systemic therapies among NSCLC patients for the purpose of informing medical recommendations and taking effective medical decisions. There are many factors that can be used as an effective prognostic factor for immunotherapy, including smoking status, body mass index (BMI), a high-intensity statin regimen, and tumor cell expression of programmed death-1 (PD-L1) (8–11). In addition, inflammation and inflammatory processes have a substantial role in the development and progression of tumors, significantly affecting treatment effectiveness among patients with cancer (12). In recent years, researchers have developed various inflammation-related prognostic models, such as the neutrophil to lymphocyte ratio (NLR), the Glasgow prognostic score, the modified Glasgow prognostic score (GPS/mGPS), the tumor immune dysfunction and exclusion (TIDE) score, the Gustave Roussy immune (GRIm)-score, the lung immuno-oncology prognostic score (LIPS-3), and the lung immune prognostic index (LIPI). Wang et al. indicated that baseline NLR can be regarded as a prognostic biomarker for NSCLC patients taking systemic therapy (13), and a higher GPS has been shown to correlate with poor clinical outcomes (14). The TIDE dysfunction score, which is related to tumor immune evasion, is an effective indicator of immunotherapy response and resistance (15). The GRIm-score is based on patients’ NLR and albumin serum levels, and GRIm-score at 45 days since the patients’ first pembrolizumab injection has been shown to be an effective biomarker of clinical outcomes in NSCLC (16). LIPS-3 is a prognostic classification of patients receiving first-line PD-1 inhibitor for PD-L1 ≥ 50% NSCLC (17). Previous studies have indicated that LIPI can predict clinical outcome across many tumor types, such as renal cell carcinoma, melanoma, small-cell lung cancer, and especially NSCLC (18, 19).

The LIPI is defined according to a derived neutrophil to lymphocyte ratio (dNLR; absolute neutrophil count/[white blood cell (WBC) count − absolute neutrophil count]) greater than 3 as well as lactate dehydrogenase (LDH) levels greater than the upper limit of normal (ULN); LIPI is divided into three groups (good: 0 factors; intermediate: 1 factor; poor: 2 factors) (20), patients with 1 or 2 factors were considered as having a higher LIPI score compared to patients with 0 factors. The index is validated, easily derived from blood assays, and can be implemented to stratify patients based on their prognostic factor within clinical practice.

With regard to the LIPI, the number of relevant studies examining the prognostic value of this factor has increased since Mezquita et al. conducted a study exploring the prognostic value of the LIPI in NSCLC patients undergoing immunotherapy and chemotherapy (20). These authors investigated the associations between the LIPI and lung cancer survival outcomes among patients with different pathological types and according to different treatment regimens. However, the prognostic value of the LIPI in NSCLC patients remains a divisive issue. It was reported in a Japanese cohort that the LIPI was merely a valuable prognostic factor for specific subsets of NSCLC (21). Hence, this study aims to conduct a retrospective study at our center and perform a meta-analysis to provide the accurate prognostic value of the LIPI in NSCLC patients.

## Materials and Methods

### Patient Cohorts

#### Immunotherapy-Treated Cohort

This retrospective study enrolled NSCLC patients presenting at the Department of Respiratory and Critical Care Medicine at the Affiliated Jinling Hospital who were treated with immune checkpoint inhibitors between May 2017 to April 2019. In total, 98 patients were enrolled. Of these, seven were excluded because of a lack of basal LDH measures that were necessary for the LIPI score calculation (**Supplementary Figure 1**).

#### Chemotherapy and Targeted Therapy Cohorts

Of the 1,510 patients initially diagnosed with advanced NSCLC at Jinling Hospital from January 2015 to December 2019, 899 were selected for our study. Exclusion criteria covered the following: insufficient data for compiling the LIPI (130 patients with no basal LDH measures and 16 patients with no basal dNLR measures), receiving other therapies such as anti-angiogenic therapy and radiotherapy, and loss to follow-up or incomplete follow-up. The final cohort included 329 patients receiving targeted therapy (36.6%) and 570 patients receiving chemotherapy (63.4%) (**Supplementary Figure 2**).

The LIPI is divided into three groups (good: 0 factors; intermediate: 1 factor; poor: 2 factors) (20), patients with 1 or 2 factors were considered as having a higher LIPI score compared to patients with 0 factors.

We examined progression-free survival (PFS) and overall survival (OS) to evaluate the clinical outcome of NSCLC patients. PFS is defined as the period from the start of anti-tumor treatment to the progression of disease (according to the Response Evaluation Criteria in Solid Tumors (RECIST) version 1.1). OS is delimited as the time from the beginning of the treatment to death for any reason. The study was conducted in accordance with the principles of the Declaration of Helsinki (as revised in 2013).

### Literature Search

We conducted a systematic review of the PubMed and Embase databases, using the search terms “(((Lung Immune Prognostic Index[Text Word]) OR (LIPI[Text Word])) OR (LIPI score[Text Word])) AND (((((((((((((((((((Neoplasia[Title/Abstract]) OR (Neoplasias[Title/Abstract])) OR (Neoplasm[Title/Abstract])) OR (Tumors[Title/Abstract])) OR (Tumor[Title/Abstract])) OR (Cancer[Title/Abstract])) OR (Cancers[Title/Abstract])) OR (Malignancy[Title/Abstract])) OR (Malignancies[Title/Abstract])) OR (Malignant Neoplasms[Title/Abstract])) OR (Malignant Neoplasm[Title/Abstract])) OR (Neoplasm, Malignant[Title/Abstract])) OR (Neoplasms, Malignant[Title/Abstract])) OR (Benign Neoplasms[Title/Abstract])) OR (Neoplasms, Benign[Title/Abstract])) OR (Benign Neoplasm[Title/Abstract])) OR (Neoplasm, Benign[Title/Abstract]))) OR (“Neoplasms”[Mesh]))” and also included references from relevant articles (that were not identified in the database search). The database search was conducted on September 11, 2020. The included articles were subject to a dual review by two authors, and references in the identified manuscripts were reviewed manually for any additional publications. Our team also searched the PROSPERO database without restrictions, and no articles were found. The registration number is CRD42020209009.

### Quality Assessment and Data Extraction

All the included studies had to meet the following criteria: (1) Patients diagnosed with NSCLC by histopathological analysis; (2) baseline LIPI score was graded and recorded before systemic therapy; (3) endpoints including PFS or OS were reported; and (4) survival estimates were present in the form of HRs with 95% confidence intervals (CIs) or Kaplan–Meier curves. Data identified through the above criteria were independently collected by two authors (XC and LH). Any problems with data extraction were resolved through a team discussion. We collected the following data: author’s name, year of publication, country, therapy type, number of participants (i.e., number of patients with good, intermediate, and poor LIPI scores, respectively), as well as disease outcomes (PFS/OS and associated hazard ratios [HRs] and 95% confidence intervals [CI]). For studies that did not report HR values, we derived corresponding survival estimates from Kaplan-Meier curves using the Engauge Digitizer software.

### Statistical Analysis

χ2 or Fisher exact tests were used to compare the clinical characteristics between different LIPI groups. Survival estimates were calculated by the Kaplan-Meier method, and comparison between subgroups was performed by the log-rank test. Univariate Cox regression analysis was implemented to analyze HRs and associated confidence intervals. To compare survival estimates among the three groups, a pairwise comparison was conducted to correct for multiplicity.

For the meta-analysis, we computed the weighted average PFS or OS reported for patients with different LIPI groups. In addition, the team examined I² statistics as well as the p-values of each result to assess heterogeneity between articles. If I^2^ ≤ 50%, we used a random-effects model; otherwise, a fixed-effects model was chosen. A p-value less than 0.05 was considered to indicate statistically significant heterogeneity. A subgroup analysis was performed based on the distinction of treatment. Begger’s funnel plot was used to assess publication bias in this meta-analysis. Sensitivity analysis was performed to assess the robustness of the meta-analysis. SPSS 23.0 and STATA version 15 were used for all statistical analyses. All p-values were two-sided, and a p-value less than 0.05 was defined as the threshold of statistical significance.

## Results

### Results in Our Cohort

#### Patient Characteristics

Among all patients receiving immunotherapy, a total of 91 were included in the final analysis. Among these 91 patients, 61 patients (67.0%) had a good LIPI score, 23 patients (25.3%) had an intermediate LIPI score, and 7 patients (7.7%) had a poor LIPI score. Patient demographics and clinical data are shown in **Table 1**. Among patients receiving chemotherapy, a total of 59.6% (n = 340), 32.5% (n = 185), and 7.9% (n = 45) presented with good, intermediate, and poor LIPI scores, respectively. For patients receiving targeted therapy, 64.1% (n = 211), 28.3% (n = 93), and 7.6% (n = 25) had good, intermediate, and poor LIPI scores, respectively. **Supplementary Table 1** illustrates the clinical baseline and demographic features of these patients.

**Table 1:** Baseline demographic and clinical characteristics of NSCLC patients receiving immunotherapy.

Characteristics	Total LIPI (SD/IQR) n=91	Good LIPI n=61	Intermediate LIPI n=23	Poor LIPI n=7	p-value
**Median age (years)**	62.4 (32-83)				
<65		35 (57.4)	11 (47.8)	5 (71.4)	0.51
≥65		26 (42.6)	12 (52.2)	2 (28.6)	
**Sex**					
Male	67 (73.6)	44 (72.1)	19 (82.6)	4 (57.1)	0.367
Female	24 (26.4)	17 (27.9)	4 (17.4)	3 (42.9)	
**Performance status**					
0-1	59 (64.8)	38 (62.3)	16 (69.6)	5 (71.4)	0.767
≥2	32 (35.2)	23 (37.7)	7 (30.4)	2 (28.6)	
**Smoking status**					
Former/current	40 (44.0)	33 (54.1)	10 (43.5)	5 (71.4)	0.403
Never	51 (56.0)	28 (45.9)	13 (56.5)	2 (28.6)	
**Histology**					
Adenocarcinoma	55 (60.4)	37 (60.7)	14 (60.9)	4 (57.1)	0.317
Squamous cell carcinoma	33 (36.3)	21 (34.4)	9 (39.1)	3 (42.9)	
Other	3 (3.3)	3 (4.9)	0 (0.0)	0 (0.0)	
**Therapy line**					
1st	31 (34.1)	22 (36.1)	6 (26.1)	3 (42.9)	0.412
2nd	26 (28.6)	20 (32.8)	5 (21.7)	1 (14.3)	
≥3rd	34 (37.4)	19 (31.1)	12 (52.2)	3 (42.9)	
**Actionable drivers**					
Yes	29 (31.9)	17 (27.9)	9 (39.1)	3 (42.9)	0.777
*EGFR mutation*	11	4	6	1	
*ALK rearrangement*	3	3	0	0	
*KRAS*	6	5	0	1	
*Others*	9	5	3	1	
No	38 (41.7)	28 (45.9)	8 (34.8)	2 (28.6)	
Unknown	24 (26.4)	16 (26.2)	6 (26.1)	2 (28.6)	

ALK, anaplastic lymphoma kinase; EGFR, epidermal growth factor receptor; IQR, interquartile range; KRAS, Kirsten rat sarcoma viral oncogene homolog; LIPI, lung immune prognostic index; SD, standard deviation.

#### Associations Between LIPI Score and PFS in NSCLC Patients Receiving Systemic Therapy

**Figure 1:**
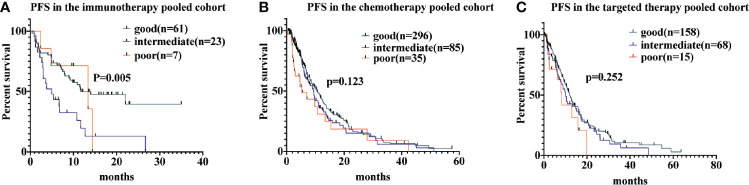
Associations of the lung immune prognostic index (LIPI) score and progression-free survival (PFS) following systemic therapy among non-small cell lung carcinoma (NSCLC) patients. Kaplan-Meier curves of PFS, comparing patients receiving immunotherapy, chemotherapy, and targeted therapy, are presented in **(A–C)**, respectively.

Among patients receiving immunotherapy, the median PFS for those with a good LIPI score was 11.7 months (95% CI, 2.6-20.7 months), while for those with a intermediate LIPI score was 5.0 months (95% CI, 2.1-7.8 months) and for those with a poor LIPI score was 13.4 months (95% CI, 0.8-25.9 months); hence, PFS differed significantly based on LIPI groups (P = 0.005). Univariate analyses showed that HRs were 2.5 (95% CI, 1.4−4.5) and 1.26 (95% CI, 0.4−3.5) for those with intermediate and poor LIPI scores, respectively, compared to those with a good LIPI score. As for patients receiving chemotherapy, median PFS for those with poor, intermediate, and good LIPI scores was 5.3 months, 9.4 months, and 10.6 months, respectively (P = 0.123). In the targeted therapy cohort, the median PFS was 8.3 months (95% CI, 5.6−10.9 months), 10.1 months (95% CI, 13.1−18.4 months), and 12.0 months (95% CI, 22.0−30.7 months) for those with poor, intermediate, and good LIPI scores, respectively (P = 0.252; **Figure 1**). Results of pairwise comparison of PFS among the three LIPI groups are shown in **Supplementary Table 2**.

#### Associations Between LIPI Score and OS in NSCLC Patients Receiving Systemic Therapy

**Figure 2:**
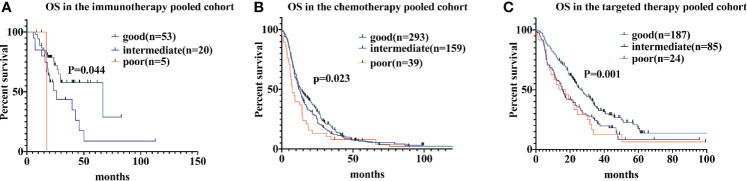
Associations of the lung immune prognostic index (LIPI) score and overall survival (OS) following systemic therapy among non-small cell lung carcinoma (NSCLC) patients. Kaplan-Meier curves of OS, comparing patients receiving immunotherapy, chemotherapy, and targeted therapy, are presented in **(A–C)**, respectively.

For patients receiving immunotherapy, median OS was 17.4 months, 26.2 months, and 66.7 months for those with poor, intermediate, and good LIPI scores, respectively (P = 0.044). Univariate analyses indicated that the HR was 2.2 (95% CI, 1.1−4.4) for the intermediate group and 3.1 (95% CI, 0.69−14.1) for the poor group. For patients receiving chemotherapy, median OS was 7.5 months, 12.1 months, and 13.3 months for those with poor, intermediate, and good LIPI scores, respectively (P = 0.023); we found HRs of 1.1 (95% CI, 0.9−1.4) and 1.6 (95% CI, 1.1−2.2) for the intermediate and poor LIPI group, respectively. Finally, for those receiving targeted therapy, the median OS for those with poor, intermediate, and good LIPI scores was 13.6 months, 15.8 months, and 26.4 months, respectively. Compared to those with a good LIPI score, a higher LIPI score was associated with worse OS, with associated HRs of 1.6 (95% CI, 1.2−2.2) and 1.8 (95% CI, 1.1−2.7), respectively (**Figure 2**). Results of pairwise comparison of OS among the three LIPI groups are shown in **Supplementary Table 3**.

### Meta-Analysis

#### Results of Searching Strategy

**Figure 3:**
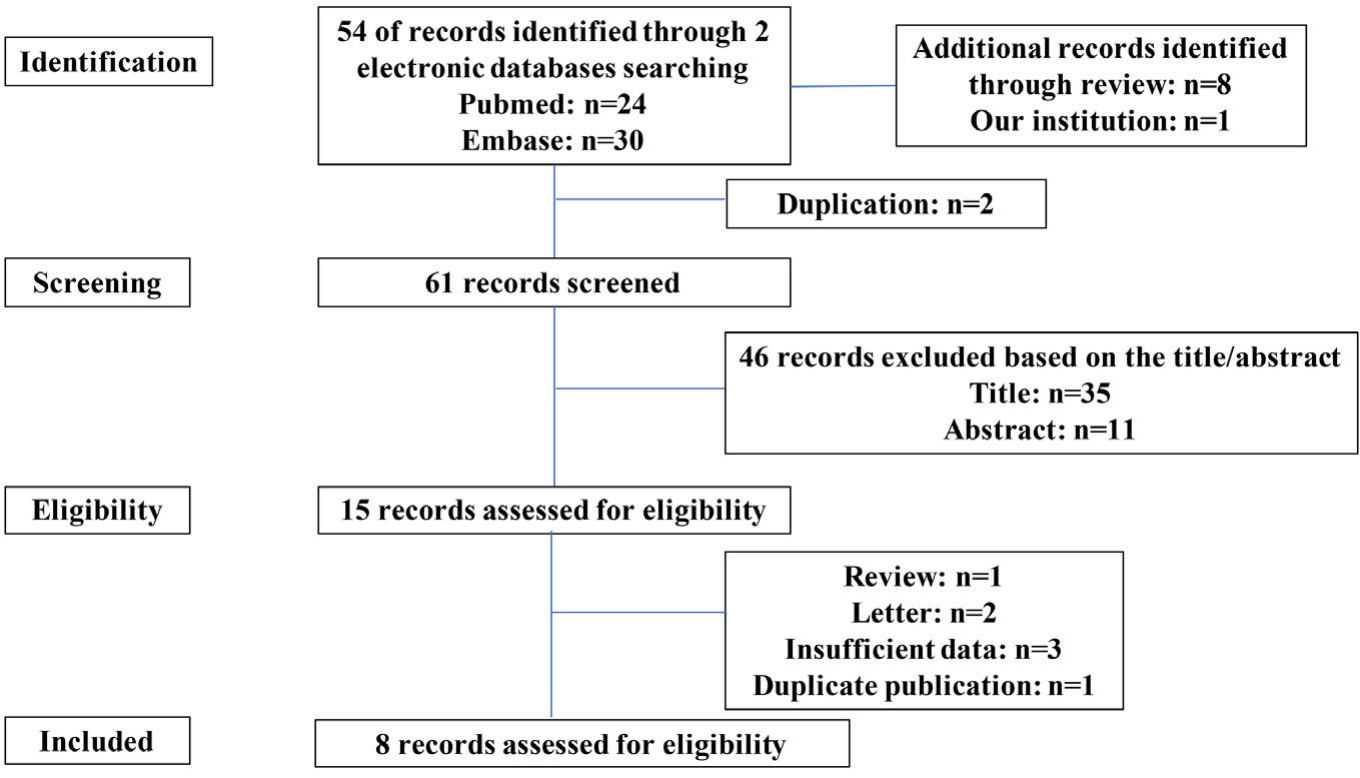
Literature search and study selection procedures.

A total of 54 relevant reports were retrieved from the search databases, and eight additional studies were identified through reviewing the reference sections of these manuscripts. After a screening and eligibility assessment, 15 studies were examined with a full-text screening. Of these 15 studies, we excluded six reports, including one review, two letters, three incomplete studies, and one duplication. Thus, eight studies (including our cohort) were suitable for the quantitative analysis (**Figure 3**).

#### Description of Studies

All reports were retrospective studies. Eight of these studies assessed immunotherapy, five articles reported on chemotherapy, and three articles reported targeted therapy. A total of 8,721 patients from the studies met our inclusion criteria. Among these patients, 45.2% (n = 3943) received immune checkpoint inhibitors, 36.8% (n = 3208) received chemotherapy, and 18.0% (n = 1570) received targeted therapy. The population distributions and characteristics of the included studies are presented in **Table 2**. HRs for PFS and OS were available within four articles, and HRs for the other four articles were extracted using Kaplan-Meier curves.

**Table 2:** Baseline characteristics of included studies.

Study	Country	Patients (n)	Tumor type	Therapy	LIPI (n)	PFS (HR 95% CI)	OS (HR 95% CI)
Mezquita (20)	France/Spain	431	NSCLC	ICI	Good (162)	Good (1)	Good (1)
					Intermediate (206)	Intermediate (1.3 95% CI 1.0-1.6)	Intermediate (1.4 95% CI 1.1-1.8)
					Poor (63)	Poor (2.3 95% CI 1.7-3.1)	Poor (2.8 95% CI 1.9-4.1)
		157		Chemo	Good (53)	Good (1)	Good (1)
					Intermediate (70)	Intermediate (1.0 95% CI 0.7-1.4)	Intermediate (1.2 95% CI 0.8-1.7)
					Poor (34)	Poor (1.3 95% CI 0.8-2.1)	Poor (1.9 95% CI 1.1-3.2)
Kazandjian (22)	US	1368	NSCLC	ICI	Good (620)	Good (1)	Good (1)
					Intermediate (583)	Intermediate (1.3 95% CI 1.1-1.4)	Intermediate (1.6 95% CI 1.4-1.9)
					Poor (165)	Poor (1.6 95% CI 1.4-2.0)	Poor (3.0 95% CI 2.4-3.8)
		1072		Chemo	Good (380)	Good (1)	Good (1)
					Intermediate (508)	Intermediate (1.2 95% CI 1.1-1.4)	Intermediate (1.3 95% CI 1.1-1.6)
					Poor (184)	Poor (1.7 95% CI 1.4-2.0)	Poor (2.0 95% CI 1.6-2.5)
		1110		TT	Good (524)	Good (1)	Good (1)
					Intermediate (424)	Intermediate (1.3 95% CI 1.1-1.5)	Intermediate (1.7 95% CI 1.4-2.1)
					Poor (162)	Poor (2.1 95% CI 1.7-2.6)	Poor (3.6 95% CI 2.7-4.8)
		437		Chemo	Good (162)	Good (1)	Good (1)
					Intermediate (191)	Intermediate (1.2 95% CI 0.9-1.5)	Intermediate (1.1 95% CI 0.8-1.5)
					Poor (84)	Poor (1.7 95% CI 1.3-2.3)	Poor (2.6 95% CI 1.8-4.0)
Meyers (19)	Canada	302	NSCLC	ICI	Good (124)	Good (1)	Good (1)
					Intermediate (124)	Intermediate (1.4 95% CI 1.0-1.8)	Intermediate (1.6 95% CI 1.1-2.1)
					Poor (54)	Poor (3.0 95% CI 2.1-4.4)	Poor (4.1 95% CI 2.8-6.0)
Minami (21)	Japan	175	WT-NSCLC	Chemo	Good (85)	Good (1)	Good (1)
					Intermediate (68)	Intermediate (1.0 95% CI 0.7-1.4)	Intermediate (1.4 95% CI 1.0-2.1)	
					Poor (22)	Poor (1.3 95% CI 0.7-2.3)	Poor (1.6 95% CI 0.9-2.9)	
		131	EGFR+	TT	Good (69)	Good (1)	Good (1)	
					Intermediate (52)	Intermediate (1.5 95% CI 1.0-2.4)	Intermediate (2.3 95% CI 1.3-3.9)	
					Poor (10)	Poor (2.6 95% CI 1.1-6.0)	Poor (2.7 95% CI 1.0-7.4)	
		110	SCC	Chemo	Good (61)	Good (1)	Good (1)	
					Intermediate (39)	Intermediate (1.0 95% CI 0.6-1.7)	Intermediate (1.0 95% CI 0.6-1.6)	
					Poor (10)	Poor (2.0 95% CI 0.9-4.2)	Poor (1.6 95% CI 0.7-3.4)	
Ruiz-Bañobre (23)	Spain	153	NSCLC	ICI	Good (77)	Good (1)	Good (1)	
					Intermediate (63)	Intermediate (1.6 95% CI 1-2.5)	Intermediate (3.1 95% CI 1.4-7.0)	
					Poor (13)	Poor (3.2 95% CI 1.5-7.1)	Poor (7.5 95% CI 2.2-25.5)	
Sorich (24)	Australia	1489	NSCLC	ICI	Good (678)	Good (1)	Good (1)	
					Intermediate (631)	Intermediate (1.4 95% CI 1.2-1.6)	Intermediate (1.7 95% CI 1.4-2.0)	
					Poor (180)	Poor (2.0 95% CI 1.7-2.4)	Poor (3.9 95% CI 3.1-4.8)	
		687		Chemo	Good (290)	Good (1)	Good (1)	
					Intermediate (328)	Intermediate (1.3 95% CI 1.1-1.5)	Intermediate (1.5 95% CI 1.2-1.8)	
					Poor (69)	Poor (2.0 95% CI 1.5-2.7)	Poor (2.8 95% CI 2.1-3.8)	
Mazzaschi (25)	Italy	109	NSCLC	ICI	NR	Good (1)	Good (1)	
Huang (2021)	China	91	NSCLC	ICI	Good (61)	Intermediate (2.6 95% CI 1.3-5)	Intermediate (2 95% CI 0.9-4.1)	
						Poor (4.5 95% CI 2.1-9.6)	Poor (4.8 95% CI 1.6-14.3)	
						Good (1)	Good (1)	
					Intermediate (23)	Intermediate (2.5 95% CI 1.4-4.5)	Intermediate (2.2 95% CI 16.0-36.3)	
					Poor (7)	Poor (1.2 95% CI 0.4-3.5)	Poor (3.1 95% CI 0.6-14.0)	
		570		Chemo	Good (340)	Good (1)	Good (1)	
					Intermediate (185)	Intermediate (1.1 95% CI 0.8-1.4)	Intermediate (1.1 95% CI 0.9-1.3)	
					Poor (45)	Poor (1.5 95% CI 1.0-2.4)	Poor (1.1 95% CI 1.0-2.2)	
		329		TT	Good (211)	Good (1)	Good (1)	
					Intermediate (93)	Intermediate (1.1 95% CI 0.8-1.6)	Intermediate (1.6 95% CI 1.1-2.1)	
					Poor (25)	Poor (1.6 95% CI 0.8-3.1)	Poor (1.7 95% CI 1.1-2.7)	

NSCLC, non-small cell lung cancer; WT, wild-type; EGFR, epidermal growth factor receptor; SCC, squamous cell carcinoma; ICI, immune checkpoint inhibitor; Chemo, chemotherapy; TT, targeted therapy; LIPI, lung immune prognostic index; PFS, progression-free survival; HR, hazard ratio; CI, confidence interval; OS, overall survival.

#### Associations Between LIPI Score and PFS in NSCLC Patients Receiving Systemic Therapy

**Figure 4:**
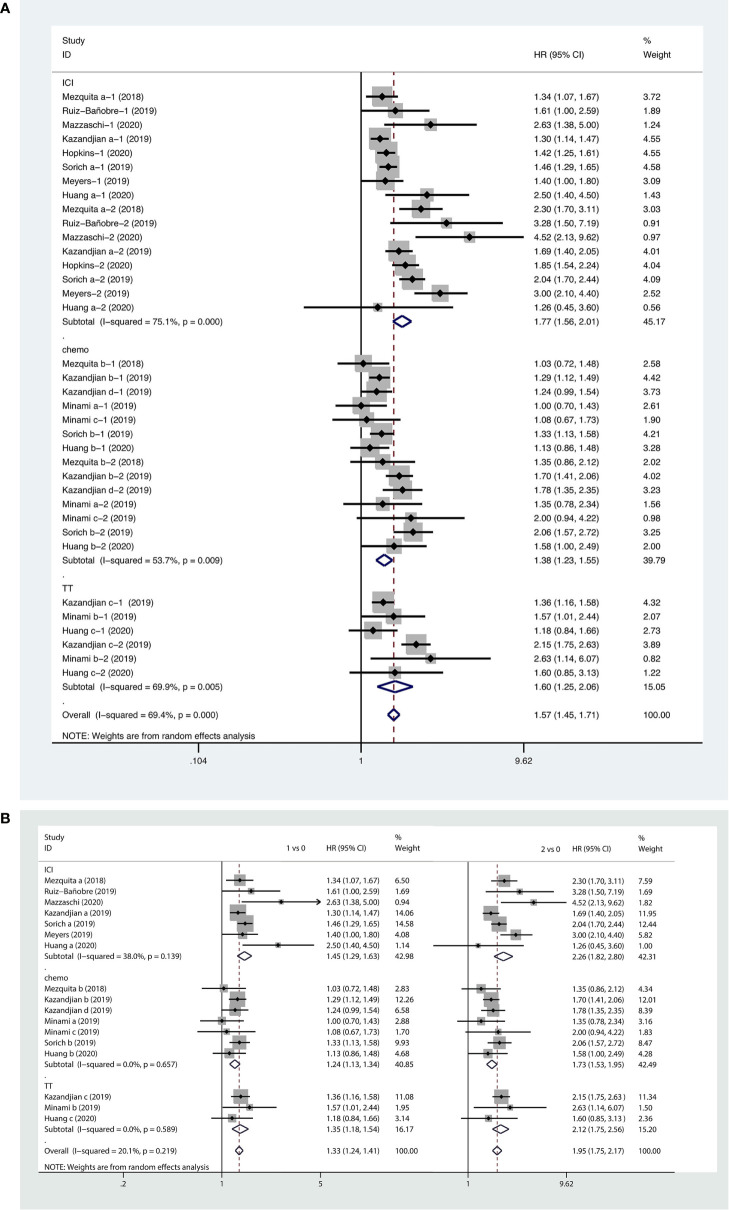
Forest plots of pooled studies examining associations between the lung immune prognostic index (LIPI) score and progression-free survival (PFS), with subgroup analyses categorized by treatment region **(A)** and LIPI scores **(B)**; HR, hazard ratio; CI, confidence interval; LIPI score of 1 vs. 0: intermediate versus good; LIPI score of 2 vs. 0: poor versus good.

Based on this meta-analysis, we found that a higher LIPI score was associated with shorter PFS (HR, 1.57; 95% CI, 1.45−1.71). The forest plot showed that worse PFS was observed in patients with poor LIPI (HR, 1.95; 95% CI, 1.75−2.17) and intermediate LIPI scores (HR, 1.33; 95% CI, 1.24−1.41) compared to patients with good LIPI scores. Subgroup analyses on the basis of therapeutic strategy demonstrated the prognostic value of a higher LIPI score harbored in all systemic therapies, including immunotherapy (HR, 1.77; 95% CI, 1.56−2.01), chemotherapy (HR, 1.38; 95% CI, 1.23−1.55), and targeted therapy (HR, 1.60; 95% CI, 1.25−2.06). The pooled results of the meta-analysis are shown in **Figure 4**.

#### Association Between LIPI Score and OS in NSCLC Patients Receiving Systemic Therapy

**Figure 5:**
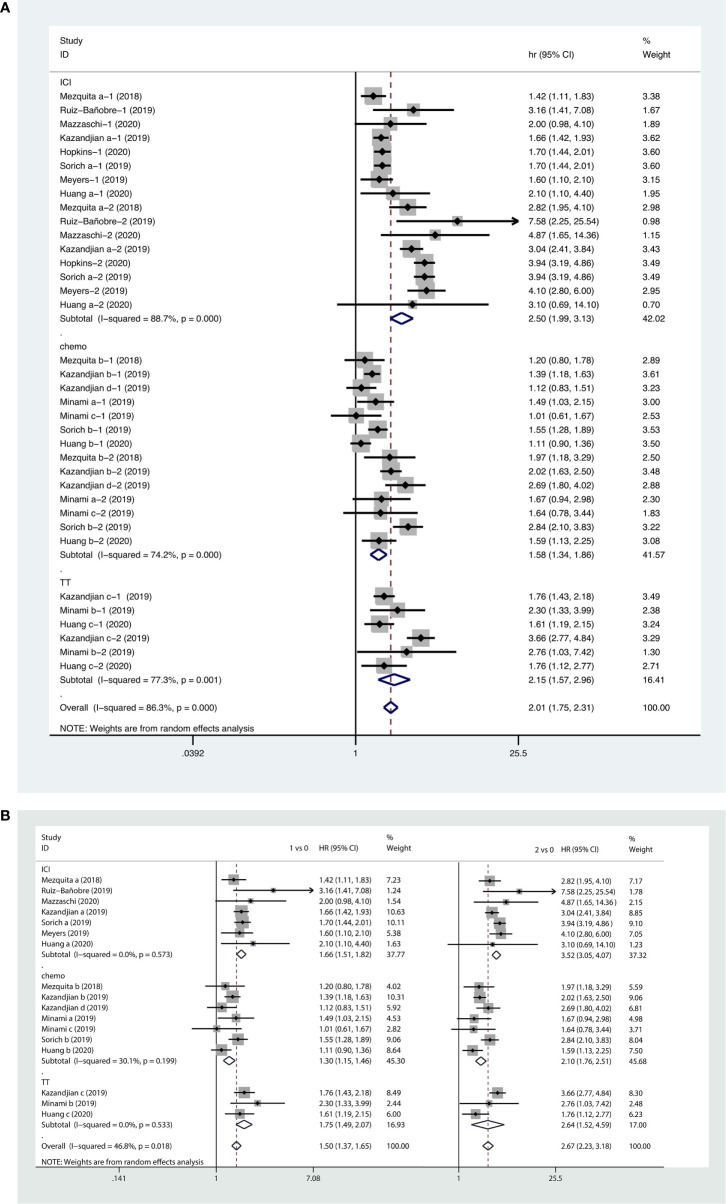
Forest plots of pooled studies examining associations between the lung immune prognostic index (LIPI) score for overall survival (OS), with subgroup analyses categorized by treatment region **(A)** and LIPI scores **(B)**; HR, hazard ratio; CI, confidence interval; LIPI score of 1 vs. 0: intermediate versus good; LIPI score of 2 vs. 0: poor versus good.

Forest plots of the pooled results showed that higher LIPI score (HR, 2.01; 95% CI, 1.75−2.31), as well as intermediate (HR, 1.52; 95% CI, 1.37−1.65) and poor LIPI scores (HR, 2.67; 95% CI, 2.23−3.18) were risk factors for poor OS in NSCLC patients receiving systemic therapy. Subgroup analyses showed that a higher LIPI score was associated with worse clinical outcomes with regard to all systemic therapies, including immunotherapy (HR, 2.50; 95% CI, 1.99−3.13), chemotherapy (HR, 1.58; 95% CI, 1.34−1.86), and targeted therapy (HR, 2.15; 95% CI, 1.57−2.96) (**Figure 5**).

#### Publication Bias Analysis and Sensitivity Analysis

Funnel plots with regard to OS and PFS are shown in the **Supplementary Materials**. The results indicated no publication bias (all p-values > 0.05) (**Supplementary Figure 3**). We conducted sensitivity analysis for the OS and PFS outcomes, and the pooled results demonstrated that removing any one study in any order did not influence our results, which indicated that the results were stable and reliable (**Supplementary Figure 4**).

## Discussion

In our cohort, we found that a higher LIPI score was a prognostic factor for OS and PFS in NSCLC patients receiving immunotherapy and for OS in patients receiving chemotherapy or targeted therapy. However, we found no association between LIPI score and PFS in NSCLC patients receiving chemotherapy or targeted therapy.

With regard to the meta-analysis, the pooled results of eight studies (which included 8,721 patients) demonstrated that baseline LIPI score was associated with the survival outcomes of NSCLC patients receiving systemic therapy through various treatment regimens. Subgroup analyses indicated that a higher LIPI score was related to inferior efficacy of immunotherapy, chemotherapy, as well as targeted therapy.

An uncontrolled inflammatory reaction is one of the main mechanisms underlying the occurrence of malignant tumors. DNA damage caused by long-term chronic inflammation can lead to the development of cancer (26, 27). An excessive proliferation of lung epithelial cells can lead to the secretion of various inflammatory cytokines, chemokines, and other inflammatory mediators (28, 29). Proto-oncogene products activate inflammatory pathways (30), increase DNA damage, and accelerate cell aging, thereby further aggravating tumor inflammation; this in turn generates a cascade of immunosuppressive cells, such as bone marrow-derived immune inhibiting tumor cells and tumor-associated macrophages (31). Thus, inflammation is closely related to the occurrence and development of tumors. To date, researchers have investigated the relationship between WBCs, neutrophils, monocytes, platelets, dNLR, C-reaction protein (CRP), and other systemic inflammatory reaction indicators and the progression/treatment efficacy of NSCLC (32).

The dNLR is an effective indicator of systemic inflammation (33). Elevated dNLR levels may indicate an angiogenic or pro-inflammatory state in the tumor microenvironment, which reflects the balance between neutrophils and lymphocytes, as well as the patient’s overall immune status. LDH is a key enzyme involved in tumor metabolism and is a biomarker of poor survival in patients with cancer. It is capable of accelerating resistance to systemic therapy by promoting immune suppression and immune escape (34, 35).

Evaluating a combination of multiple prognostic factors has great advantages in clinical practice. At present, many researchers are committed to identifying valuable biomarkers to predict the efficacy of systemic treatment in NSCLC, especially with regard to immunotherapy (36). However, there is currently a lack of effective factors. The LIPI can be used to assess systemic inflammation among patients based on dNLR and LDH levels and can help to evaluate the level of systemic inflammation in patients with cancer. Our results indicate that a higher LIPI score is related to worse clinical outcome among NSCLC patients receiving systemic therapy. Both dNLR and LDH are routine and objective indicators that can be accessed without difficulty during clinical practice; each hospital has a uniform standard to define abnormal laboratory testing results.

Our study was the first to investigate the prognostic value of the LIPI among Chinese NSCLC patients receiving systemic therapy, and our results regarding target therapy were different from those of previous studies in Western countries. Of note, the most common mutation in our cohort was the epidermal growth factor receptor (*EGFR*) gene mutation, which is the most frequent driver gene mutation in NSCLC; this mutation has a prevalence of 10% in the Western NSCLC population, but the prevalence is as high as 50% in the Asian NSCLC population (37). The relationship between LIPI score and PFS of NSCLC patients with target therapy might be different between different populations and ethnic groups, so more data from Asian populations are needed in the future.

To our knowledge, only Xie et al. have published a meta-analysis about the relationship between the LIPI and efficacy of different treatments in NSCLC patients (38). In this article, there were only four studies included. Meanwhile, the article only included overall survival as outcome indicators. Our meta-analysis included more studies, especially the study performed in our institution, which is the first analysis about the association between LIPI score and PFS/OS of NSCLC patients in the Chinese cohort. Including our cohort of Chinese patients added patient diversity and improved the external interpretation of the meta-analysis. Therefore, we have provided stronger evidence concerning the prognostic value of LIPI score in advanced NSCLC patients.

This study presents limitations as well as strengths. Our study was a retrospective, single-center study. Although we conducted a meta-analysis, all of the included studies were retrospective studies. Moreover, baseline characteristics including pathological classification were not available in most of the literature, preventing further subgroup analysis in specific subsets of NSCLC patients. Specifically, research on targeted therapy cohorts mainly incorporated information on *EGFR* and anaplastic lymphoma kinase (*ALK*); other driver mutations should be considered in future studies. Additionally, in our immunotherapy cohort, the median PFS of the poor LIPI group were even longer than those of the good and intermedia group, and the intermediate group was the one with the worst prognosis. The reason may lie in that the sample size of the poor LIPI group was small (n=7). In fact, all the patients in the poor LIPI group had developed disease progression before the 14th month, whereas the other two groups had not. This phenomenon could be directly caused by limited patients in the poor group. Thus, these relevant results should be cautiously interpreted. Further studies with larger cohorts are required to verify our results. Besides, results from the meta-analysis with a large sample in the poor group (n=551) suggested that poor LIPI score was associated with worse prognoses in NSCLC patients. Moreover, some survival information was unavailable; therefore, digitizing and extracting Kaplan-Meier curve-related survival information through the Engauge Digitizer might have inevitably led to some bias.

## Conclusions

In summary, among advanced NSCLC patients receiving systemic therapy, a higher baseline LIPI score (i.e., an intermediate or poor LIPI score) was associated with poorer survival outcomes. Additional randomized clinical trials are required to comprehensively inquire into the predictive value of the LIPI in NSCLC.

## Data Availability Statement

The original contributions presented in the study are included in the article/**Supplementary Material**. Further inquiries can be directed to the corresponding authors.

## Author Contributions

LH and LZ conceived the study. LH, XC, and QX authored the article. JX performed the literature review and extracted the signature models. LH, HH, PZ, and SC performed the analyses and wrote the first draft of the manuscript, supported by TL and YS. All authors contributed to the article and approved the submitted version.

## Funding

This work was supported by the Jiangsu Provincial Social Development - Key Projects - Clinical Frontier Technologies (grant number BE2019719) and the Jiangsu Provincial Social Development - General Program (grant number BE2019718).

## Conflict of Interest

The authors declare that the research was conducted in the absence of any commercial or financial relationships that could be construed as a potential conflict of interest.
