# Supplementary material for: Evaluation of the Lung Immune Prognostic Index in Non-Small Cell Lung Cancer Patients Treated With Systemic Therapy: A Retrospective Study and Meta-Analysis
Source: Front Oncol. 2021 Jun 24;11:670230. doi: 10.3389/fonc.2021.670230 (PMC8264771; doi:10.3389/fonc.2021.670230)

Supplementary Material

Supplementary Table 1 Baseline demographic and clinical characteristics of NSCLC patients with targeted therapy and chemotherapy.

**Characteristics**	**Targeted Therapy N=329 (%)**	**Chemotherapy N=570 (%)**
Age(years), median range	60.7 （26.0~86.0）	61.0 (26.0~84.0)
Gender		
Male	133 (40.4)	391 (68.6)
Female	196 (59.6)	179 (31.4)
Smoking status		
Former-smoker	25 (7.6)	82 (14.4)
Current-smoker	60 (18.2)	193 (33.9)
Non-smoker	244 (74.2)	295 (51.8)
Histology		
Adenocarcinoma	310 (94.2)	413 (72.5)
Squamous cell carcinoma	6 (1.8)	135 (23.7)
Other	13 (4.0)	22 (3.9)
EGFR		
Wild type	43 (13.1)	387 (67.9)
Mutation	259 (78.7)	80 (14.0)
19	124 (47.9)	41 (51.1)
21	125 (48.3)	26 (32.5)
Other	10 (3.9)	13 (16.3)
Unknown	27 (8.2)	103 (18.1)
LIPI		
Good	211 (64.1)	340 (59.6)
Intermediate	93 (28.3)	185 (32.5)
Poor	25 (7.6)	45 (7.9)

Supplementary Table 2: Pairwise comparison of Progression-free survival (PFS) in three LIPI groups.

	Immunotherapy(*P*-value)	Target therapy(*P*-value)	Chemotherapy(*P*-value)
Good vs Intermedia	0.002	0.431	0.332
Good vs Poor	0.666	0.142	0.056
Intermedia vs Poor	0.199	0.251	0.118

Supplementary Table 3: Pairwise comparison of Overall survival (OS) in three LIPI groups.

	Immunotherapy(*P*-value)	Target therapy(*P*-value)	Chemotherapy(*P*-value)
Good vs Intermedia	0.07	0.001	0.284
Good vs Poor	0.213	0.01	0.013
Intermedia vs Poor	0.473	0.764	0.065

Supplementary figure

Supplementary figure 1: Flow chart of our targeted immunotherapy cohort.
Supplementary figure 2: Flow chart of our targeted therapy and chemotherapy cohort.
Supplementary figure 3: Funnel plot of progression-free survival; 3A: funnel plot of all including studies; funnel plot of subgroup analysis 3B (LIPI: good vs intermediate) and 3C (LIPI: good vs poor)
Supplementary figure 4: Funnel plot of Overall survival; 4A: funnel plot of all including studies; funnel plot of subgroup analysis 4B (LIPI score: good vs intermediate) and 4C (LIPI score: good vs poor)
Supplementary figure 5: Sensitivity analysis of progression-free survival (5A) and overall survival (5B)

Supplementary figure 1

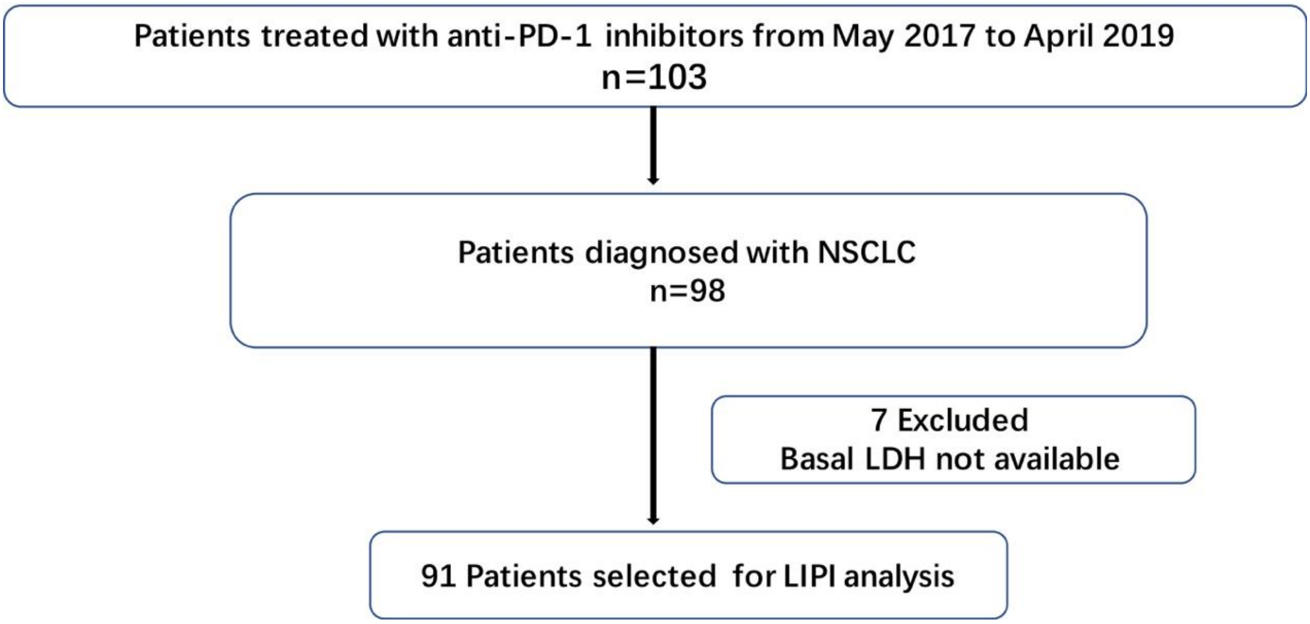

Supplementary figure 2

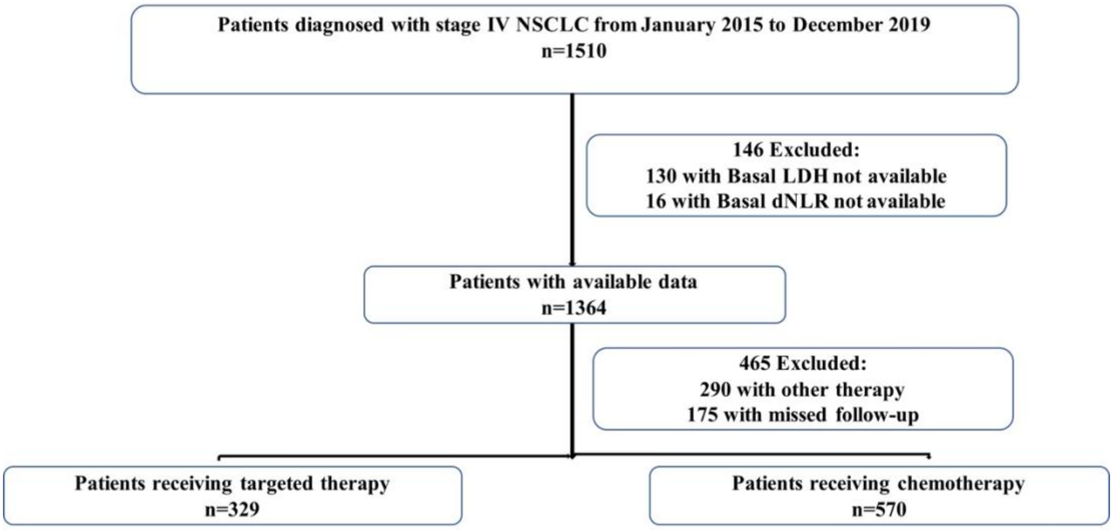

Supplementary figure 3A

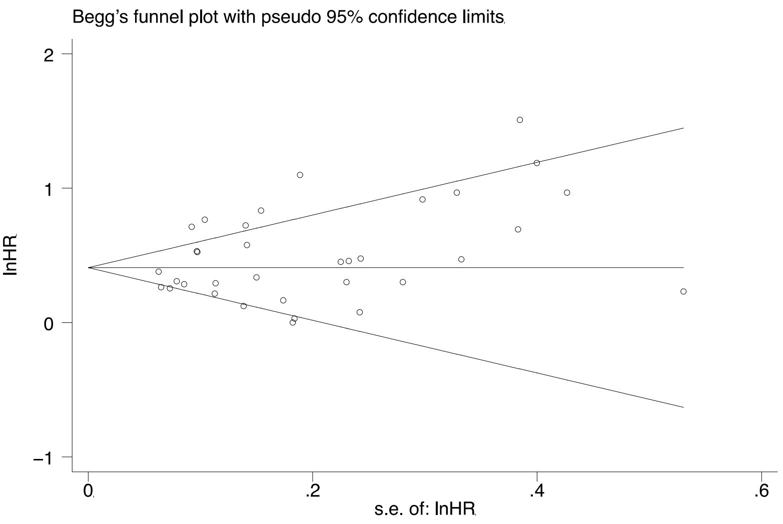

Supplementary figure 3B

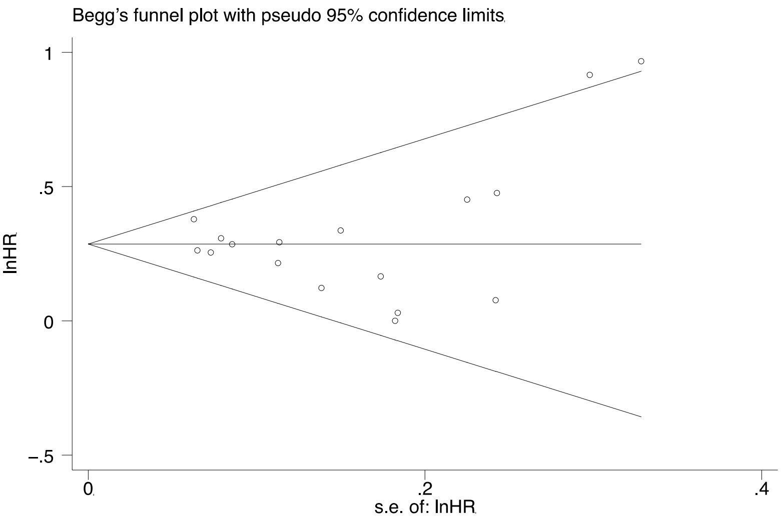

Supplementary figure 3C

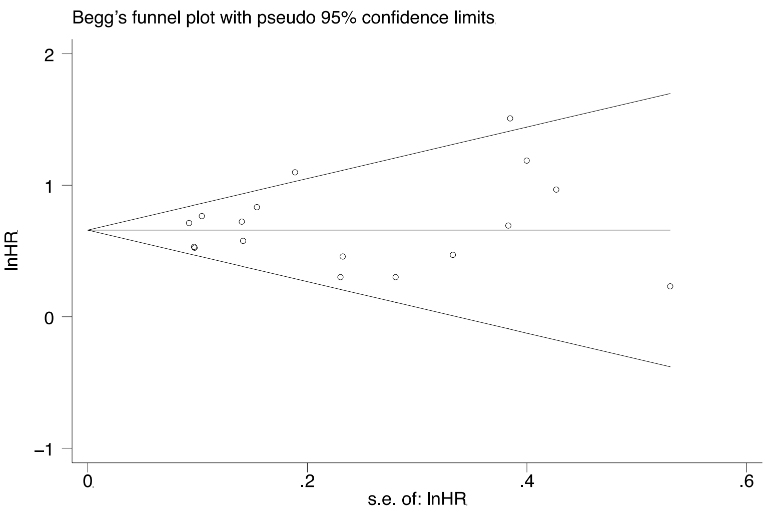

Supplementary figure 4A

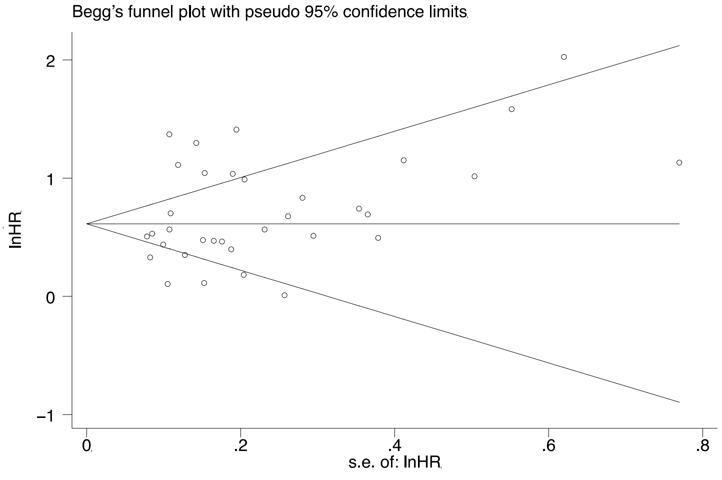

Supplementary figure 4B

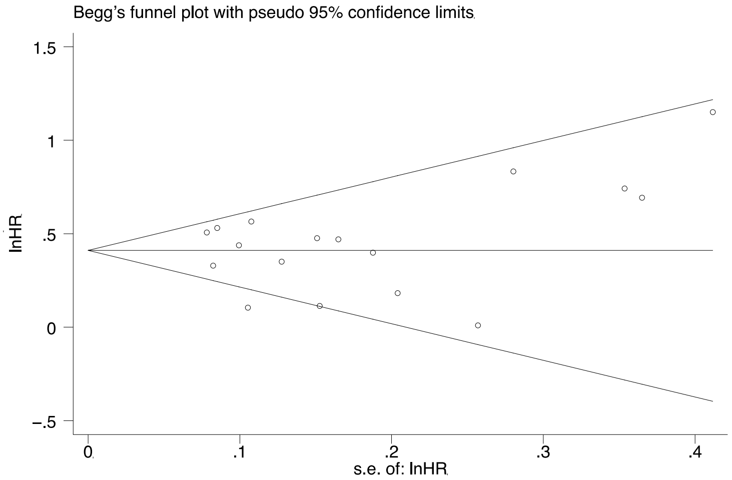

Supplementary figure 4C

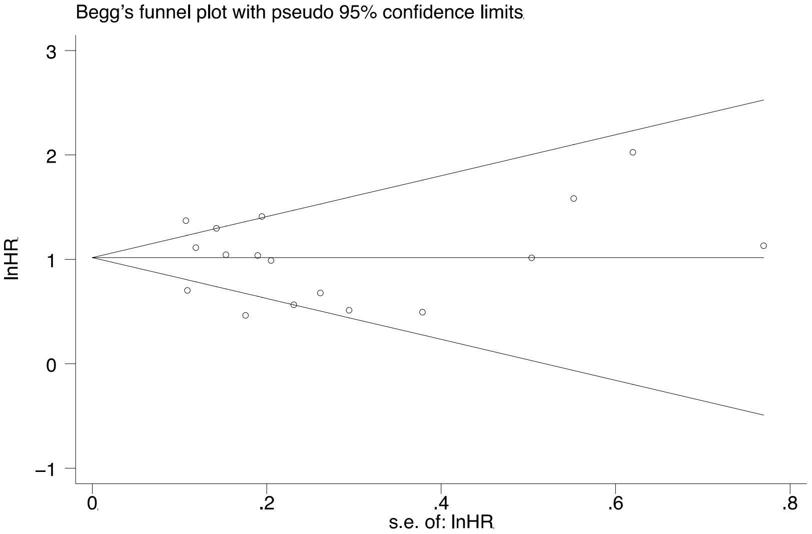

Supplementary figure 5A

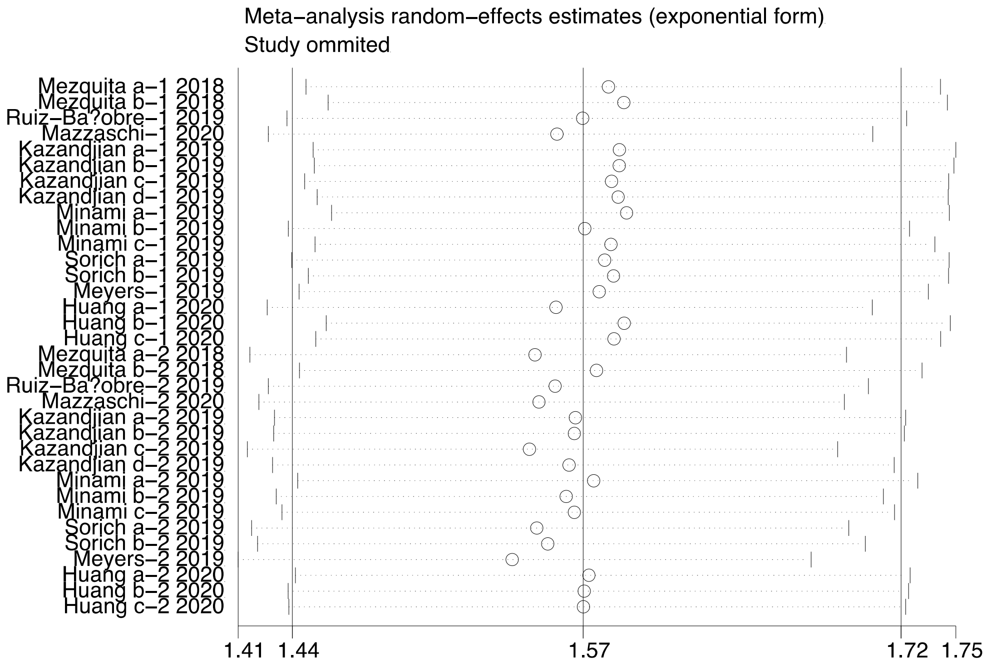

Supplementary figure 5B

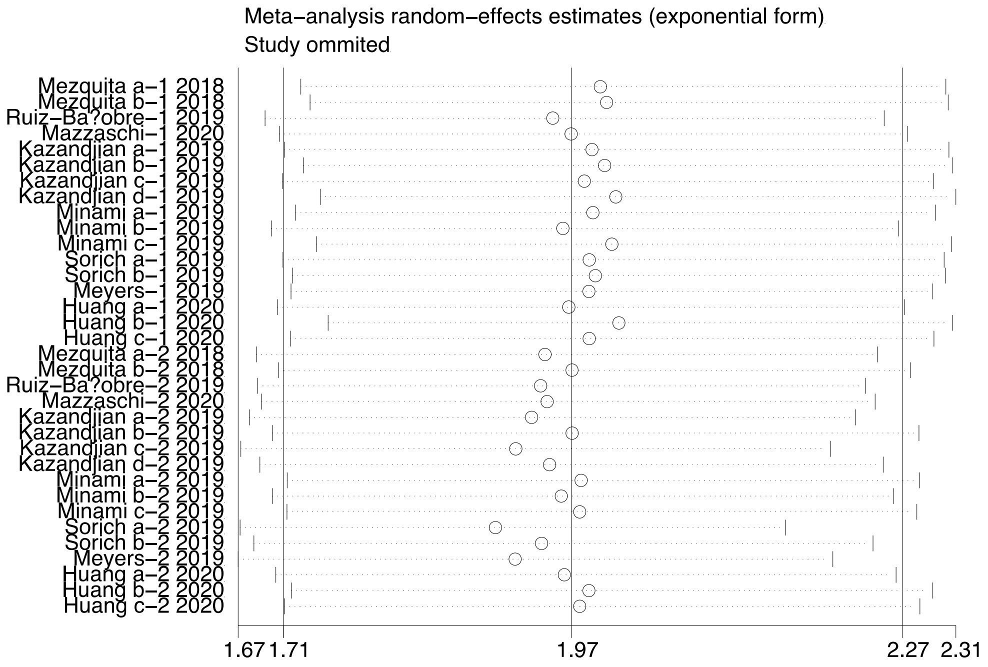

Supplement: Supplementary file 1 [file DataSheet_1.pdf]
